# How Accurate Is Multiple Imputation for Nutrient Intake Estimation? Insights from ASA24 Data

**DOI:** 10.3390/nu17152510

**Published:** 2025-07-30

**Authors:** Nicolas Woods, Jason Gilliland, Louise W. McEachern, Colleen O’Connor, Saverio Stranges, Sean Doherty, Jamie A. Seabrook

**Affiliations:** 1 School of Health Studies, Western University, London, ON N6A 3K7, Canada; nwoods@uwo.ca (N.W.); jgillila@uwo.ca (J.G.); 2 Human Environments Analysis Laboratory, Western University, London, ON N6A 3K7, Canada; lmceach4@uwo.ca (L.W.M.); sdoherty@wlu.ca (S.D.); 3 Children’s Health Research Institute, London, ON N6C 2V5, Canada; 4 Department of Geography and Environment, Western University, London, ON N6A 5C2, Canada; 5 Department of Epidemiology and Biostatistics, Western University, London, ON N6G 2M1, Canada; saverio.stranges@uwo.ca; 6 Department of Paediatrics, Western University, London, ON N6A 5W9, Canada; 7 Lawson Research Institute, London, ON N6A 4V2, Canada; colleen.oconnor@uwo.ca; 8 London Health Sciences Centre Research Institute, London, ON N6A 5W9, Canada; 9 Brescia School of Food and Nutritional Sciences, Western University, London, ON N6G 2V4, Canada; 10 Department of Family Medicine, Western University, London, ON N6G 2M1, Canada; 11 Department of Medicine, Western University, London, ON N6A 5A5, Canada; 12 Department of Clinical Medicine and Surgery, University of Naples Federico II, 80131 Naples, Italy; 13 Department of Geography and Environmental Studies, Wilfrid Laurier University, Waterloo, ON N2L 3C5, Canada

**Keywords:** multiple imputation, nutrient intake estimation, 24 h recalls, missing data, dietary assessment tools, nutritional epidemiology, implausible dietary recalls, adolescent nutrition, nutrient thresholds, data imputation accuracy

## Abstract

**Background/Objectives**: Accurate dietary assessment is crucial for nutritional epidemiology, but tools like 24 h recalls (24HRs) face challenges with missing or implausible data. The Automated Self-Administered 24 h Dietary Assessment Tool (ASA24) facilitates large-scale data collection, but its lack of interviewer input may lead to implausible dietary recalls (IDRs), affecting data integrity. Multiple imputation (MI) is commonly used to handle missing data, but its effectiveness in high-variability dietary data is uncertain. This study aims to assess MI’s accuracy in estimating nutrient intake under varying levels of missing data. **Methods**: Data from 24HRs completed by 743 adolescents (ages 13–18) in Ontario, Canada, were used. Implausible recalls were excluded based on nutrient thresholds, creating a cleaned reference dataset. Missing data were simulated at 10%, 20%, and 40% deletion rates. MI via chained equations was applied, incorporating demographic and psychosocial variables as predictors. Imputed values were compared to actual values using Spearman’s correlation and accuracy within ±10% of true values. **Results**: Spearman’s rho values between the imputed and actual nutrient intakes were weak (mean ρ ≈ 0.24). Accuracy within ±10% was low for most nutrients (typically < 25%), with no clear trend by missingness level. Diet quality scores showed slightly higher accuracy, but values were still under 30%. **Conclusions**: MI performed poorly in estimating individual nutrient intake in this adolescent sample. While MI may preserve sample characteristics, it is unreliable for accurate nutrient estimates and should be used cautiously. Future studies should focus on improving data quality and exploring better imputation methods.

## 1. Introduction

Accurately measuring dietary intake in individuals is inherently challenging. Common dietary assessment methods include food diaries, food frequency questionnaires [1], and emerging technologies such as photographic records [2]. Among these, the 24 h dietary recall (24HR) is one of the most widely used tools and is designed to provide a detailed account of all foods and beverages consumed on the previous day [1].

Unlike other dietary assessment methods, the 24HR typically relies on a trained interviewer to elicit detailed information. For instance, when a participant reports consuming “a bagel,” the interviewer may follow up with specific questions about the type (e.g., whole grain vs. refined flour), flavour (e.g., plain vs. cinnamon raisin), brand, preparation method (e.g., toasted or not), condiments (e.g., butter, cream cheese), accompanying beverages, and portion size [1]. Although this level of detail allows for more accurate nutrient analysis [3], it also increases respondent burden and the cognitive demand of recall.

While trained interviewers enhance the accuracy of dietary recalls, they also represent a key limitation of traditional 24HRs [4]. Since these interviews are typically conducted one-on-one [3], larger-scale studies must hire multiple interviewers or extend the study duration to accommodate all participants. Each 24HR can take up to an hour to complete [3], making the use of 24HRs a resource-intensive method. As a result, researchers often turn to alternatives such as food frequency questionnaires (FFQs), which are self-administered, less time-consuming, and require minimal training [5]. However, despite their practicality, FFQs are less accurate than 24HRs in estimating specific nutrient intakes [4], limiting their suitability for certain types of analyses.

To address the time and resource burden associated with traditional 24HRs, the National Cancer Institute developed the Automated Self-Administered 24 h Dietary Assessment Tool (ASA24) [6]. The ASA24 utilizes the Automated Multiple-Pass Method (AMPM) to collect detailed information about respondents’ dietary intake from the previous day. Conducted entirely online, ASA24 is well-suited for large-scale nutrition research [6]. By eliminating the need for trained interviewers, ASA24 overcomes many logistical challenges of traditional 24HRs, enabling multiple participants to complete recalls simultaneously and making it feasible for use in large epidemiological studies.

Despite its advantages, the absence of an interviewer in ASA24 introduces new limitations. Participants may misreport their intake—either unintentionally, through typographical errors or misinterpretation of prompts, or intentionally, through selective reporting—resulting in implausible dietary recalls (IDRs) [7]. Many of these errors might have been identified and corrected during interviewer-led 24HRs, but in automated settings, they can distort both mean and variability estimates of nutrient intake. Although ASA24 incorporates standardized portion size prompts and facilitates anomaly detection during data cleaning [8], the removal or exclusion of IDRs still represents a loss of potentially valuable data.

Missing data can reduce statistical power, increase the risk of Type II errors [9,10,11], and bias study results, particularly when the missing data are systematically different from those observed [12]. While researchers may commonly use listwise deletion to address this issue, this approach can exacerbate bias [13]. A more sophisticated approach is multiple imputation (MI), which replaces missing values with plausible estimates derived from fitted models across multiple newly created datasets [14]. MI is particularly effective when data are Missing Completely at Random (MCAR) or Missing at Random (MAR)—that is, when the probability of missingness is unrelated or explainable by observed variables [15].

Nevertheless, all imputation methods—including MI—share a fundamental limitation: they involve estimation without knowing the true values. While MI is more robust than simpler approaches, its accuracy in the context of high-variability outcomes like daily nutrient intake remains unclear [15,16]. Given the day-to-day fluctuation in dietary behaviours (and subsequent nutrient intake), it is essential to understand how well MI can reconstruct missing dietary data, particularly in large-scale nutrition studies that use ASA24.

To date, no study has directly evaluated the accuracy of MI in estimating missing nutrient intake data derived from 24HRs. Therefore, the overall goal of this study was to assess the performance of MI in accurately reconstructing nutrient intake values under conditions of simulated missingness. To do so, the following specific objectives were identified:

1. To assess the correlation between imputed and true values using Spearman’s rho at 10%, 20%, and 40% levels of simulated missing data.
2. To evaluate the accuracy of imputed values, defined as being within ± 10% of the actual value for each nutrient.
3. To examine trends in correlation strength and accuracy across increasing proportions of missing data.

## 2. Materials and Methods

### 2.1. Study Design and Data Source

Data for this study were drawn from the SmartAPPetite for Youth Study, a cluster-randomized controlled trial conducted in Southwestern Ontario, Canada, among adolescents aged 13–18 years from 2017 to 2020. This age range was selected because it corresponds to the standard age span for high school students in Ontario, Canada, which was the intended target population for the SmartAPPetite intervention. That study aimed to evaluate a smartphone application (“SmartAPPetite”) intended to improve food knowledge, food purchasing, and diet quality [17]. Relevant to this current study, the SmartAPPetite for Youth participants completed two tools at three time points—baseline, post-intervention, and follow-up. The tools were (1) a 24 h dietary recall using ASA24, and (2) a “youth survey” that assessed dietary habits and related psychosocial factors.

A formal sample size calculation was not performed for this secondary analysis, which was based on pre-existing data collected during the SmartAPPetite for Youth cluster-randomized trial. While the original study was powered to detect intervention effects on dietary outcomes, the current analytic sample of 743 adolescents is sufficiently large to support a robust evaluation of multiple imputation accuracy across varying levels of simulated missingness.

### 2.2. Measures

#### 2.2.1. ASA24 Dietary Recall

The ASA24 dietary recall aimed to capture participants’ dietary intake for the previous 24 h. This validated tool follows a 7-step AMPM process [18] inquiring about foods consumed and associated mealtimes; a probe for additional foods not previously reported; detailed questions about food items, including preparation method, portion size, brand, and condiments; review and editing of entered data; prompting for commonly forgotten foods (e.g., snacks consumed while commuting or shopping); final confirmation of entries; and a self-assessment of whether the reported intake reflected usual intake. Upon completion, ASA24 automatically calculates nutrient intakes using its internal food composition database.

While ASA24 may slightly underestimate the intake of certain nutrients (e.g., energy, protein) when compared to recovery biomarkers [19], it demonstrates comparable accuracy to traditional 24HRs in estimating nutrient intake [19,20,21]. This makes ASA24 a practical and reliable tool for use in large-scale epidemiological studies.

#### 2.2.2. Youth Survey

The youth survey included questions on demographics (e.g., age, sex, ethnicity), self-reported physical and mental health, food-related behaviours and general eating habits (e.g., allergies, cooking frequency, meal skipping), perceived importance of healthy eating, and food purchasing behaviours. Participants were also asked to provide their primary residence’s postal code, which was used to calculate median neighbourhood income. A food knowledge quiz, adapted from two validated instruments [22,23], was administered at the end of the survey.

#### 2.2.3. Nutrient and Diet Quality Measures

From the full nutrient output generated by ASA24, the following 21 nutrients were selected for analysis based on their relevance and previous epidemiologic research: calories (kcal), protein (g), total fat (g), saturated fat (g), carbohydrates (g), total sugars (g), fibre (g), calcium (mg), iron (mg), magnesium (mg), potassium (mg), sodium (mg), zinc (mg), vitamin C (mg), thiamin (mg), riboflavin (mg), niacin (mg), folate (mcg), vitamin B12 (mcg), and vitamin A (mcg, RAE). Two composite diet quality scores were also calculated: Healthy Eating Index-2015 (HEI-2015) [24,25] and Nutrient Rich Foods Index 9.3 (NRF 9.3) [26].

### 2.3. Additional Covariates

Variables from the youth survey included in the analysis were sex, age, ethnicity (White/Caucasian: yes/no), self-rated physical and mental health, number of physically active days in the past week (0–7 days), perceived importance of eating healthy, and total food knowledge score (ranging from 0 (minimum)–50 (maximum)). Additionally, a proxy for socioeconomic status was considered by incorporating a variable for neighbourhood-level median household income, as calculated by linking each participant’s primary residence’s postal code to 2016 Canadian census data at the dissemination area level [27,28]. Information on how each question from the youth survey was asked can be found in Appendix A.

### 2.4. Data Cleaning and Identification of Implausible Dietary Recalls

To identify IDRs, thresholds were applied to ASA24-derived nutrient intakes based on established guidelines [8]. Specifically, records were set to “missing” if any of the following nutrient values fell outside plausible ranges (Table 1):

These thresholds were derived from the upper and lower 5% bounds of National Health and Nutrition Examination Survey (NHANES) data distributions [8].

### 2.5. Simulation of Missing Data

Following the creation of a cleaned dataset—one with no missing entries and no implausible nutrient values—a simulation procedure was implemented to artificially introduce missing data. A random number generator was used to randomly select dietary records to be set as missing. For each selected case, the corresponding dietary intake data were exported and stored separately as the reference “true” values. These original values were then removed from the dataset to simulate realistic patterns of missingness.

The dataset was subsequently prepared for MI. An MI model using chained equations was fitted, generating 200 imputed datasets. Predictor variables included in the imputation model were sex, age, white/Caucasian ethnicity (yes/no), self-reported physical health, self-reported mental health, number of physically active days in the past week, perceived importance of healthy eating, total food knowledge score, and neighbourhood-level median household income.

Once imputation was completed, the average of the 200 values was calculated for each nutrient and used as the “final” imputed estimate. The original (true) values were then reinserted into the dataset for comparative analysis.

### 2.6. Analysis of Imputation Data

To assess the performance of the imputation model, comparisons were made between the imputed values and their corresponding true values on an intra-individual basis. Descriptive statistics, including means and standard deviations, were computed for both actual and imputed values. Spearman’s rho was used to calculate correlation coefficients between true and imputed nutrient values, with corresponding *p*-values to determine statistical significance. A *p*-value < 0.05 was considered statistically significant.

To evaluate practical accuracy, a ±10% threshold was applied. Specifically, for each nutrient, an imputed value was considered accurate if it fell within 10% of the individual’s true value. For instance, for a participant with an actual energy intake of 2000 kcal, any imputed value between 1800 and 2200 kcal would be classified as accurate. The proportion of imputed values meeting this criterion was then calculated.

This entire process was repeated across three levels of simulated missing data: 10%, 20%, and 40%. These levels were chosen to reflect pragmatic real-world scenarios. A missing data level under 10% might not substantially affect results, while missingness exceeding 40% [29] may undermine study validity regardless of the missing data handling method (e.g., listwise deletion or multiple imputation).

## 3. Results

### 3.1. Descriptive Statistics

Table 2 presents the demographic and dietary characteristics of the sample (*n* = 743). Participants had a mean age of 15.6 (SD = 1.2) years, with 62.6% identifying as female and 68.1% identifying as White. Mean calorie intake was 1735.4 (SD = 712.2) kcal/day, with notable variability in macronutrient and micronutrient intake.

### 3.2. Correlation Between Imputed and Actual Values

Across all three levels of missing data (10%, 20%, 40%), Spearman’s rho (ρ) values between the imputed and actual nutrient values were consistently low (Table 3, Table 4 and Table 5). At 10% missingness, correlations were weakest, with rho values ranging from −0.10 to 0.31 and few statistically significant correlations. At 20% and 40% missingness, correlations modestly improved, with most nutrients demonstrating statistically significant yet weak correlations (mean ρ ≈ 0.24).

### 3.3. Accuracy of Imputed Values

**Figure 1:**
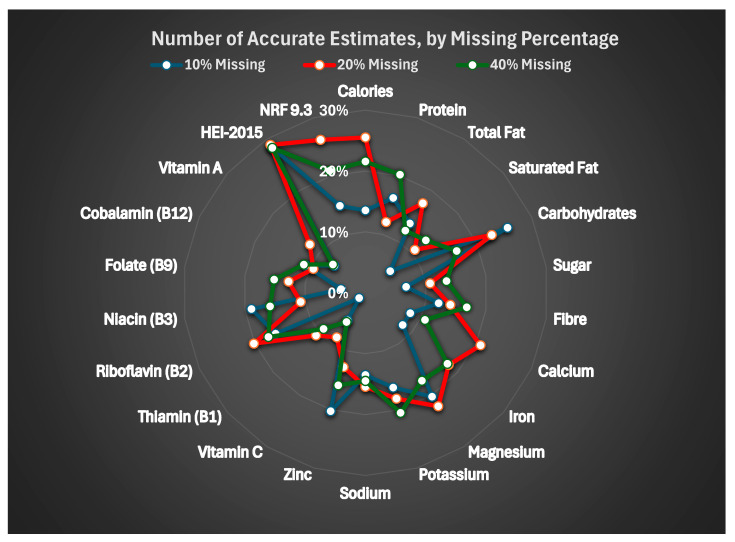
Number of accurate estimates, by missing percentage.

Table 6 and Figure 1 summarize the proportion of imputed values falling within ±10% of the actual values. Accuracy was generally poor. HEI-2015 showed the highest proportion of accurate imputation values (approximately 28%) across all levels of missingness. For nutrients, accuracy rarely exceeded 25%, and in many cases, fewer than 15% of values were within the accuracy threshold. No clear pattern of improved accuracy was observed with less missingness.

## 4. Discussion

This study evaluated the accuracy of MI for estimating missing nutrient intake data among Canadian adolescents, using 24 h dietary recall data from ASA24. To our knowledge, this is the first study to assess imputation accuracy using a reference dataset with known true values.

Across all levels of missingness, correlations between imputed and actual nutrient values were weak. While MI is regarded as a robust method when data are MAR, our findings suggest that its performance may be limited when applied to high-variability outcomes such as nutrient intake. Notably, the modest improvement in correlation coefficients at 20% and 40% missingness could be attributed to increased sample size and statistical power rather than improved model performance.

The accuracy of imputed values, defined as being within 10% of the true value, was low for most nutrients. Furthermore, and contrary to expectations, MI did not become less reliable with more missing data [30]. For example, the correlation coefficient for calories was lowest among the dataset with 10% missing data and increased as the percentage increased, while other nutrients showed no clear pattern of relationship between missing data percentage and coefficient values. Even the most accurately imputed variable (HEI-2015) had correct estimates in less than one-third of cases. These findings suggest that although MI may preserve overall distributions and allow for full sample inclusion, it does not reliably reproduce individual-level nutrient intake data. This is critical, as nutritional epidemiology research often relies on accurate intake values to examine exposure–outcome relationships.

Our findings diverge from previous research that used MI in FFQs or registry data, largely because those studies lacked true values for comparison. For example, studies from Japan [31], Italy [32], and the U.S. [33] assessed MI performance via comparisons to complete-case analyses, rather than direct validation against known intakes. While such approaches may provide insight into relative bias, they cannot assess absolute accuracy, as the missing values remain unknown.

Importantly, this study leveraged a rare opportunity to simulate missingness within a dataset of plausible recalls, thereby enabling direct comparison between imputed and true values. While this design enhances internal validity, it also introduces limitations and potential biases. The study excluded IDRs to establish a known reference, which may not reflect real-world patterns where data are often missing not at random (MNAR) [30]. Moreover, our use of a single 24 h recall per participant limited our ability to estimate usual intake, which likely constrained the imputation model’s performance. This was necessary because the source data came from an intervention study. Using multiple recalls could have altered the “usual intake” over time because of the intervention itself.

Additionally, the classification of implausible recalls based on extreme values in energy and select nutrients, while conservative and consistent with ASA24 guidance [8], may have excluded some valid recalls. We opted for this method as we did not have the necessary data to apply more refined methods such as the Goldberg cutoff [34], which would have required measured body weight or energy expenditure data.

Nevertheless, this study has important strengths. It is the first to assess the absolute accuracy of MI for nutrient data using known true values, across varying levels of missingness. It also adopts an individual-level evaluation approach, offering insights that average-based comparisons cannot. Additionally, because ASA24 allowed for multiple dietary recalls without accompanying interviewer requirements, we were able to collect a large enough sample to examine detailed dietary intakes of participants, which may not have been possible using traditional 24HRs. Our pragmatic use of ASA24 and common demographic covariates makes this study directly applicable to dietitians and other nutrition researchers conducting primary nutrition studies.

## 5. Conclusions

In conclusion, MI for missing nutrient intake data demonstrated limited accuracy when compared to known values, even when standard modelling techniques and a validated dietary assessment tool were used. These findings highlight the need for caution when using MI for individual-level dietary data and underscore the importance of improving data quality at the point of collection. Future research should explore methods to enhance imputation accuracy, consider repeated recalls for estimating usual intake, and investigate model performance in datasets with true MNAR characteristics.

## Figures and Tables

**Table 1:** Classifying implausible dietary recalls by sex.

Nutrient	Boys (Min–Max)	Girls (Min–Max)
Energy (kcal)	650–5700	600–4400
Protein (g)	10–240	10–240
Fat (g)	15–230	15–230
Vitamin C (mg)	5–400	5–400

**Table 2:** Demographic and nutrient intake information of sample (*n* = 743).

Variable	Mean ± SD, or *n* (%)
Sex (Female), *n* (%)	465 (62.6%)
Age	15.6 ± 1.2
Ethnicity (White)	506 (68.1%)
Physical Health Score (range: 1 (lowest)–5 (highest))	
1	54 (7.3%)
2	162 (21.8%)
3	301 (40.5%)
4	166 (22.3%)
5	60 (8.1%)
Mental Health Score (range: 1 (lowest)–5 (highest))	
1	60 (8.1%)
2	178 (24.0%)
3	227 (30.6%)
4	193 (26.0%)
5	85 (11.4%)
Number of days physically active in previous week	4.0 ± 1.9
Importance of Eating Healthy score (range: 1 (lowest)–5 (highest))	
1	0 (0%)
2	174 (23.4%)
3	347 (46.7%)
4	141 (19.0%)
5	81 (10.9%)
Nutritional Knowledge Score (range: 0–50)	29.9 ± 7.1
Median Neighbourhood-level Income (CAD)	94,507 ± 27,925
Nutrients	
Calories (kcal)	1735.4 ± 712.2
Protein (g)	76.8 ± 39.4
Total Fat (g)	66.0 ± 33.8
Saturated Fat (g)	22.0 ± 13.6
Carbohydrates (g)	212.3 ± 97.3
Sugar (g)	81.5 ± 53.6
Fibre (g)	16.0 ± 9.0
Calcium (mg)	778.4 ± 495.0
Iron (mg)	12.1 ± 5.9
Magnesium (mg)	255.1 ± 124.2
Potassium (mg)	2320.0 ± 1155.3
Sodium (mg)	2942.7 ± 1521.7
Zinc (mg)	10.0 ± 6.3
Vitamin C (mg)	90.1 ± 80.6
Thiamin (B1) (mg)	1.6 ± 0.9
Riboflavin (B2) (mg)	1.6 ± 0.8
Niacin (B3) (mg)	21.6 ± 11.6
Folate (B9) (mcg)	311.2 ± 161.5
Cobalamin (B12) (mcg)	3.6 ± 3.2
Vitamin A (mcg)	596.3 ± 511.4
HEI-2015 Score (range: 0–100)	55.3 ± 14.4
NRF 9.3 Score (range: 0–700)	369.3 ± 135.3

**Table 3:** Comparison of 10% imputed values to actual values (*n* = 74).

	Actual Values	Imputed Values	Spearman’s rho (ρ)	*p* Value
	Mean ± SD	Mean ± SD		
Calories (kcal)	1752.7 ± 851.1	1735.2 ± 242.8	0.17	0.16
Protein (g)	76.1 ± 38.1	77.0 ± 15.0	0.06	0.61
Total Fat (g)	68.8 ± 39.8	65.9 ± 10.9	0.13	0.29
Saturated Fat (g)	22.6 ± 17.3	22.2 ± 4.3	0.05	0.68
Carbohydrates (g)	212.2 ± 116.4	213.1 ± 23.2	0.23	0.05
Sugar (g)	78.9 ± 57.1	82.0 ± 10.2	0.10	0.40
Fibre (g)	16.0 ± 10.5	15.9 ± 2.0	0.20	0.08
Calcium (mg)	741.6 ± 641.2	786.1 ± 152.1	−0.01	0.93
Iron (mg)	11.5 ± 6.5	12.2 ± 1.9	0.12	0.31
Magnesium (mg)	246.4 ± 132.3	254.7 ± 34.8	0.10	0.40
Potassium (mg)	2248.8 ± 1177.2	2311.6 ± 317.3	0.07	0.56
Sodium (mg)	2979.2 ± 1860.8	2942.2 ± 516.3	0.17	0.16
Zinc (mg)	9.6 ± 5.6	10.1 ± 2.2	−0.06	0.60
Vitamin C (mg)	84.4 ± 89.3	88.6 ± 16.6	0.26	0.03 *
Thiamin (B1) (mg)	1.5 ± 0.9	1.6 ± 0.3	0.23	0.05
Riboflavin (B2) (mg)	1.5 ± 0.8	1.6 ± 0.3	0.01	0.95
Niacin (B3) (mg)	21.9 ± 11.2	21.6 ± 3.8	0.16	0.18
Folate (B9) (mcg)	294.3 ± 181.5	311.6 ± 38.1	0.26	0.03 *
Cobalamin (B12) (mcg)	3.3 ± 2.5	3.7 ± 1.0	−0.10	0.41
Vitamin A (mcg) Equivalent	541.6 ± 548.0	594.6 ± 73.5	−0.09	0.46
HEI 2015 Score	53.5 ± 15.3	55.1 ± 3.4	0.31	0.01 *
NRF 9.3 Score	349.0 ± 142.9	368.9 ± 35.9	0.19	0.10

Note. Mean ± standard deviation (SD) is reported to highlight variance reduction introduced by multiple imputation (MI), which may be less apparent using medians and interquartile ranges. * Indicates a statistically significant correlation between actual and imputed values at α = 0.05 based on the Spearman’s rho).

**Table 4:** Comparison of 20% imputed values to actual values (*n* = 149).

	Actual Values	Imputed Values	Spearman’s rho (ρ)	*p* Value
	Mean ± SD	Mean ± SD		
Calories (kcal)	1725.4 ± 719.9	1727.1 ± 232.7	0.33	<0.01 *
Protein (g)	79.4 ± 43.5	76.1 ± 14.4	0.31	<0.01 *
Total Fat (g)	64.5 ± 32.0	65.7 ± 9.8	0.28	<0.01 *
Saturated Fat (g)	21.1 ± 13.3	21.9 ± 3.9	0.29	<0.01 *
Carbohydrates (g)	211.1 ± 93.4	212.6 ± 24.9	0.20	<0.01 *
Sugar (g)	77.8 ± 46.3	81.7 ± 11.0	0.25	<0.01 *
Fibre (g)	16.4 ± 9.9	15.9 ± 2.4	0.25	0.02 *
Calcium (mg)	727.4 ± 440.8	780.4 ± 159.4	0.30	<0.01 *
Iron (mg)	12.3 ± 5.8	11.8 ± 2.1	0.27	<0.01 *
Magnesium (mg)	255.5 ± 123.0	254.3 ± 39.7	0.20	<0.01 *
Potassium (mg)	2368.5 ± 1122.4	2309.1 ± 333.3	0.19	0.01 *
Sodium (mg)	2874.9 ± 1493.8	2932.5 ± 517.8	0.29	<0.01 *
Zinc (mg)	10.5 ± 7.3	9.8 ± 2.0	0.26	<0.01 *
Vitamin C (mg)	99.1 ± 78.8	89.7 ± 17.9	0.02	0.45
Thiamin (B1) (mg)	1.6 ± 0.9	1.6 ± 0.3	0.21	<0.01 *
Riboflavin (B2) (mg)	1.5 ± 0.8	1.6 ± 0.3	0.35	<0.01 *
Niacin (B3) (mg)	23.2 ± 14.1	21.0 ± 3.6	0.22	<0.01 *
Folate (B9) (mcg)	310.8 ± 152.0	309.6 ± 44.5	0.20	0.01 *
Cobalamin (B12) (mcg)	4.0 ± 3.6	3.5 ± 0.9	0.26	<0.01 *
Vitamin A (mcg) Equivalent	582.0 ± 439.9	603.7 ± 72.5	0.18	<0.01 *
HEI 2015 Score	56.9 ± 14.5	55.1 ± 3.7	0.23	<0.01 *
NRF 9.3	377.6 ± 128.1	367.6 ± 42.2	0.22	<0.01 *

* Indicates statistically significant correlation between actual and imputed values at α = 0.05 (based on Spearman’s rho).

**Table 5:** Comparison of 40% imputed values to actual values (*n* = 297).

	Actual Values	Imputed Values	Spearman’s rho (ρ)	*p* Value
	Mean ± SD	Mean ± SD		
Calories (kcal)	1729.5 ± 712.1	1754.7 ± 248.6	0.22	<0.01 *
Protein (g)	75.6 ± 37.7	78.2 ± 15.2	0.27	<0.01 *
Total Fat (g)	65.6 ± 33.4	66.6 ± 11.4	0.18	<0.01 *
Saturated Fat (g)	21.8 ± 13.2	22.4 ± 4.5	0.19	<0.01 *
Carbohydrates (g)	213.8 ± 97.7	215.4 ± 24.8	0.16	<0.01 *
Sugar (g)	80.5 ± 55.0	83.7 ± 9.2	0.19	<0.01 *
Fibre (g)	15.9 ± 8.7	16.3 ± 2.1	0.20	<0.01 *
Calcium (mg)	747.2 ± 465.4	806.6 ± 171.8	0.23	<0.01 *
Iron (mg)	12.2 ± 5.5	12.1 ± 2.1	0.26	<0.01 *
Magnesium (mg)	250.7 ± 119.8	260.8 ± 36.6	0.22	<0.01 *
Potassium (mg)	2283.6 ± 1076.6	2361.2 ± 338.6	0.23	<0.01 *
Sodium (mg)	3008.4 ± 1509.2	2931.4 ± 585.5	0.22	<0.01 *
Zinc (mg)	9.9 ± 5.9	10.2 ± 2.2	0.23	<0.01 *
Vitamin C (mg)	86.3 ± 77.4	92.3 ± 17.2	0.06	0.28
Thiamin (B1) (mg)	1.6 ± 0.9	1.6 ± 0.3	0.23	<0.01 *
Riboflavin (B2) (mg)	1.6 ± 0.8	1.6 ± 0.3	0.26	<0.01 *
Niacin (B3) (mg)	21.7 ± 11.0	21.6 ± 3.8	0.28	<0.01 *
Folate (B9) (mcg)	322.5 ± 168.5	303.5 ± 47.2	0.15	0.01 *
Cobalamin (B12) (mcg)	3.6 ± 3.0	3.7 ± 1.0	0.21	<0.01 *
Vitamin A (mcg) equivalent	601.6 ± 529.6	595.7 ± 104.7	0.15	0.01 *
HEI 2015 Score	54.2 ± 14.2	56.1 ± 3.5	0.23	<0.01 *
NRF 9.3	366.8 ± 132.8	372.2 ± 41.5	0.23	<0.01 *

* Indicates statistically significant correlation between actual and imputed values at α = 0.05 (based on Spearman’s rho).

**Table 6:** Number of accurate estimates, by missing percentage.

Number of Estimates Within 10% of True Value			
Nutrient	10% Missing (*n*, %)	20% Missing (*n*, %)	40% Missing (*n*, %)
Calories	10 (13.5%)	38 (25.5%)	64 (21.5%)
Protein	12 (16.2%)	18 (12.1%)	60 (20.2%)
Total Fat	10 (13.5%)	26 (17.4%)	36 (12.1%)
Saturated Fat	4 (5.4%)	16 (10.7%)	39 (13.1%)
Carbohydrates	19 (25.7%)	34 (22.8%)	49 (16.5%)
Sugar	5 (6.8%)	16 (10.7%)	40 (13.5%)
Fibre	9 (12.2%)	21 (14.1%)	50 (16.8%)
Calcium	6 (8.1%)	31 (20.8%)	32 (10.8%)
Iron	6 (8.1%)	27 (18.1%)	53 (17.8%)
Magnesium	15 (20.3%)	33 (22.1%)	51 (17.2%)
Potassium	12 (16.2%)	27 (18.1%)	61 (20.5%)
Sodium	10 (13.5%)	23 (15.4%)	43 (14.5%)
Zinc	15 (20.3%)	19 (12.8%)	47 (15.8%)
Vitamin C	4 (5.4%)	13 (8.7%)	17 (5.7%)
Thiamin (B1)	1 (1.4%)	16 (10.7%)	27 (9.1%)
Riboflavin (B2)	12 (16.2%)	30 (20.1%)	52 (17.5%)
Niacin (B3)	14 (18.9%)	16 (10.7%)	47 (15.8%)
Folate (B9)	3 (4.1%)	19 (12.8%)	45 (15.2%)
Cobalamin (B12)	8 (10.8%)	14 (9.4%)	33 (11.1%)
Vitamin A Equivalent	5 (6.8%)	18 (12.1%)	21 (7.1%)
HEI 2015 Score	21 (28.4%)	43 (28.9%)	84 (28.3%)
NRF 9.3 Score	11 (14.9%)	39 (26.2%)	62 (20.9%)

## Data Availability

Supporting data may be provided upon request.

## Abbreviations

The following abbreviations are used in this manuscript:

24HRS	24 h Recalls
ASA24	Automated Self-Administered 24 h Dietary Assessment Tool
MI	Multiple Imputation
FFQs	Food Frequency Questionnaires
AMPM	Automated Multiple-Pass Method
IDRs	Implausible Dietary Recalls
MCAR	Missing Completely at Random
MAR	Missing at Random
MNAR	Missing Not at Random
HEI-2015	Healthy Eating Index-2015
NRF 9.3	Nutrient Rich Foods Index 9.3
NHANES	National Health and Nutrition Examination Survey

## Appendix A

Appendix A presents the youth survey questions used as covariates and how the questions were asked.

### Appendix A.1. Covariates and Source Questions

#### Appendix A.1.1. Sex

Sex was determined by asking respondents to respond to the following: “I am a (insert response),” where responses included “Male” “Female,” and “I identify as (please specify).” No participant who identified as neither male nor female had complete data, and therefore, sex was treated as a binary variable.

#### Appendix A.1.2. Age

Age was obtained by asking “What is your current age?” with responses being limited to ages 13–18.

#### Appendix A.1.3. Ethnicity

Ethnicity was determined by asking “What is your ethnicity? (Please select all that apply)” with possible responses of “White/Caucasian”, “South Asian (e.g., East Indian, Pakistani, Sri Lankan)”, “East Asian (e.g., Chinese, Japanese, Korean)”, “Middle Eastern (e.g., Egyptian, Iranian, Lebanese)”, “Latin American (e.g., Mexican, Columbian, Peruvian)”, “Indigenous (i.e., First Nations, Métis, or Inuit)”, “Black (e.g., African, Caribbean)”, and/or “Other (Please Specify)”.

#### Appendix A.1.4. Physical Health Score

Physical health was determined by asking “In general, how do you rate your own physical health?” with possible responses of “Excellent,” “Very Good,” “Good,” “Fair,” and “Poor.”

#### Appendix A.1.5. Mental Health Score

Mental health was determined by asking “In general, how do you rate your own mental health?” with possible responses of “Excellent,” “Very Good,” “Good,” “Fair,” and “Poor.”

#### Appendix A.1.6. Number of Days Physically Active in Previous Week

Physical activity was determined via the following question: “Physical activity is an activity that increases your heart rate and makes you feel out of breath some of the time. Add up all the time you spend engaged in physical activity each day. Some examples of physical activity are running, brisk walking, rollerblading, biking, dancing, skateboarding, swimming, soccer, or basketball.

Over the past 7 days, how many days were you physically active for a total of at least 60 min per day?”

#### Appendix A.1.7. Importance of Eating Healthy Score

Importance of eating healthy was determined via a Likert scale, where the following statement was asked to be ranked: “Eating healthy food is important to me,” with the options of “Strongly Agree,” “Agree,” “Neither Agree nor Disagree,” “Disagree,” and “Strongly Disagree.”
